# Site-Directed Spin Labeling Reveals Pentameric Ligand-Gated Ion Channel Gating Motions

**DOI:** 10.1371/journal.pbio.1001714

**Published:** 2013-11-19

**Authors:** Cosma D. Dellisanti, Borna Ghosh, Susan M. Hanson, James M. Raspanti, Valerie A. Grant, Gaoussou M. Diarra, Abby M. Schuh, Kenneth Satyshur, Candice S. Klug, Cynthia Czajkowski

**Affiliations:** 1 Department of Neuroscience, University of Wisconsin, Madison, Wisconsin, United States of America; 2 Department of Biophysics, Medical College of Wisconsin, Milwaukee, Wisconsin, United States of America; University of Texas at Austin, United States of America

## Abstract

Pentameric ligand-gated ion channels (pLGICs) are neurotransmitter-activated receptors that mediate fast synaptic transmission. In pLGICs, binding of agonist to the extracellular domain triggers a structural rearrangement that leads to the opening of an ion-conducting pore in the transmembrane domain and, in the continued presence of neurotransmitter, the channels desensitize (close). The flexible loops in each subunit that connect the extracellular binding domain (loops 2, 7, and 9) to the transmembrane channel domain (M2–M3 loop) are essential for coupling ligand binding to channel gating. Comparing the crystal structures of two bacterial pLGIC homologues, ELIC and the proton-activated GLIC, suggests channel gating is associated with rearrangements in these loops, but whether these motions accurately predict the motions in functional lipid-embedded pLGICs is unknown. Here, using site-directed spin labeling (SDSL) electron paramagnetic resonance (EPR) spectroscopy and functional GLIC channels reconstituted into liposomes, we examined if, and how far, the loops at the ECD/TMD gating interface move during proton-dependent gating transitions from the resting to desensitized state. Loop 9 moves ∼9 Å inward toward the channel lumen in response to proton-induced desensitization. Loop 9 motions were not observed when GLIC was in detergent micelles, suggesting detergent solubilization traps the protein in a nonactivatable state and lipids are required for functional gating transitions. Proton-induced desensitization immobilizes loop 2 with little change in position. Proton-induced motion of the M2–M3 loop was not observed, suggesting its conformation is nearly identical in closed and desensitized states. Our experimentally derived distance measurements of spin-labeled GLIC suggest ELIC is not a good model for the functional resting state of GLIC, and that the crystal structure of GLIC does not correspond to a desensitized state. These findings advance our understanding of the molecular mechanisms underlying pLGIC gating.

## Author Summary

Ligand-gated ion channels reside in the membranes of nerve and muscle cells. These proteins form channels that span the membrane, where they transduce chemical signals into changes in electrical excitability. Neurotransmitters bind to the extracellular surface of these proteins to trigger global structural rearrangements that open the channel, allowing ions to flow across the cell membrane. In the continued presence of neurotransmitters, the channels desensitize and close. Channel opening and closing regulate muscle contraction and signaling in the brain, and defects in these channels lead to a variety of diseases. While crystal structures have provided frozen snapshots of these proteins in presumed closed and open channel states, little is known about how the channels desensitize and move during actual signaling events. Here, we applied a technique to investigate the structure and local dynamics of proteins known as site-directed spin labeling to a prototypical ligand-gated channel, GLIC. We directly quantified ligand-induced motions in regions at the boundary between the binding domain (loops 2 and 9) and the channel domain (M2–M3 loop). We show that a large movement of loop 9 and an immobilization of loop 2, which rearranges the interface between the binding and channel domains, accompanies GLIC channel gating transitions into a desensitized state. These data provide new insights into the protein movements that underlie electrochemical transmission of signals between cells.

## Introduction

Chemical signaling in the brain and periphery relies on the rapid opening and closing of pentameric ligand-gated ion channels (pLGICs), which include nicotinic acetylcholine (nAChRs), serotonin-type-3 (5-HT_3_Rs), γ-aminobutyric acid-A (GABA_A_Rs), and glycine (GlyRs) receptors [1]. These receptors exist in at least three distinct, interconvertible states: resting (unliganded, closed channel), activated (liganded, open channel), and desensitized (liganded, closed channel), and the binding of agonists, antagonists, and allosteric drugs alters the equilibria between these states. Neurotransmitter binding in the extracellular ligand-binding domain triggers rapid opening of an intrinsic ion channel more than 60 Å away in the transmembrane domain of the receptor, and with prolonged neurotransmitter exposure, the channel moves into a nonconducting desensitized state. Although we know a fair amount about the structure of these receptors, the mechanisms by which the binding of neurotransmitter triggers channel opening and desensitization are still unfolding, and our understanding of the protein motions underlying these processes is limited.

**Figure 1:**
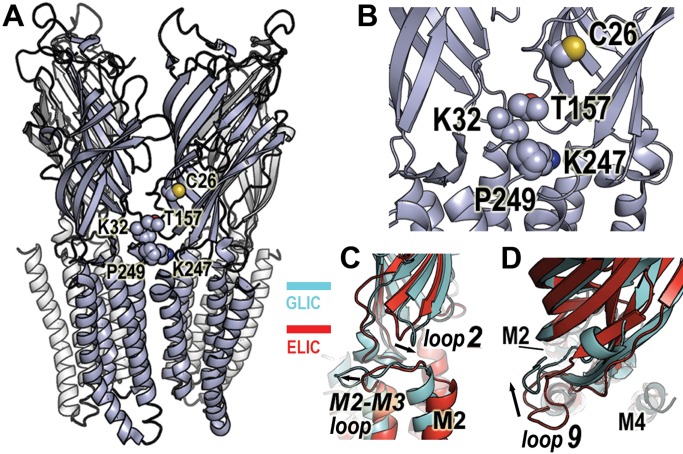
Location of loop 2, loop 9, and M2–M3 loop in GLIC. (A) Crystal structure of GLIC (PDB entry 3EHZ) with residues C26, K32, T157, K247, and P249 shown in space-fill. The fifth subunit on the backside was removed for clarity. (B) Close-up view of an intersubunit interface highlighting the region between the extracellular and transmembrane domains and the sites spin-labeled (space-fill). (C) Positions of loop 2 and M2–M3 loop in GLIC (3EHZ, cyan) and ELIC (2VL0, red) in aligned structures. Relative to ELIC, GLIC loop 2 is shifted inward towards the channel pore-lining M2 helix (labeled), whereas GLIC M2–M3 loop is shifted outward (arrows). (D) Positions of loop 9 in GLIC (cyan) and ELIC (red) in aligned structures. Relative to ELIC, GLIC loop 9 is shifted inward toward the M2 helix (arrow).

pLGICs are composed of five identical or homologous subunits arranged pseudosymmetrically around a central ion-conducting channel. Our current structural knowledge of these proteins comes from cryo-EM structures of the *Torpedo* nAChR in a presumed unliganded closed state (4 Å resolution) and liganded open state (6.2 Å resolution) [2],[3], high-resolution crystal structures of the extracellular binding domains of the nAChR α1 and α7 subunits [4],[5], crystal structures of full-length prokaryotic pLGIC homologs from *Erwinia chrysanthemi* (ELIC) and *Gloeobacter violaceus* (GLIC) solved in presumed closed and open channel conformations [6]–[8], respectively, and a recent crystal structure of a glutamate-activated chloride channel (GluCl) in an open channel conformation from *C. elegans* [9]. In general, each subunit can be divided into two parts: an extracellular binding domain (ECD) folded into a β-sandwich core and a transmembrane channel domain (TMD) consisting of four α-helical membrane-spanning segments (M1 to M4). Neurotransmitter binding occurs at sites located at interfaces between subunits in the ECD (reviewed in [1]), and the M2 helices of each of the subunits form the ion-conducting channel. In each subunit, flexible loops (loop 2, loop 7, loop 9, and the M2–M3 loop) connect the binding domain to the channel domain (Figure 1) and play a critical role in coupling binding site movements to the channel [1].

Comparison of ELIC and GLIC structures suggests that channel activation is associated with an anticlockwise concerted twist of each ECD β-sandwich and a radial tilting of the pore lining M2 α-helices away from the channel axis [7],[10]. Rearrangements in the flexible loops that form the interface between the ECD (loop 2, loop 7, and loop 9) and the TMD (M2–M3 loop) (Figure 1) are also observed. Some studies, however, suggest that the GLIC structure may correspond to a desensitized state [11],[12] and the ELIC structure to an “uncoupled” nonfunctional conformation [11],[13]. Thus, whether the motions inferred by comparing the static structures of two related (but with only 18% sequence identity) proteins solved in detergent micelles in uncertain functional states accurately predict the dynamic gating motions of a pLGIC in its native environment is unknown.

In this study, we used site-directed spin labeling (SDSL) electron paramagnetic resonance (EPR) spectroscopy and functional GLIC channels reconstituted into liposomes to examine if, and how far, the loops at the ECD/TMD gating interface move during proton-dependent gating transitions from the resting to desensitized state. SDSL EPR spectroscopy is a powerful method for monitoring the structure and dynamics of membrane proteins in conditions closely resembling the proteins' native environment [14],[15]. In SDSL EPR, a cysteine residue is introduced at a site of interest, and a sulfhydryl-specific nitroxide reagent (typically 1-oxyl-2,2,5,5-tetramethyl-3-pyrroline-3-methyl methanethiosulfonate spin label, MTSL) is covalently attached to the free sulfhydryl as a paramagnetic probe to create the R1 side chain (Figure S1A). Backbone and side chain mobility can be detected with the continuous wave (CW) method, and distances and distance changes between pairs of probes can be measured by double electron electron resonance (DEER) spectroscopy (up to ∼60 Å) [16]. SDSL EPR spectroscopy is the ideal complement to high-resolution static snapshots of crystal structures and has been used successfully to study the dynamic motions of the voltage-gated K^+^ channel [17]–[19], other membrane proteins (e.g., the mechanosensitive channel of small conductance, MscS [20], and rhodopsin [21]) and recently, GLIC [22],[23].

Here, we show that proton-dependent GLIC gating from resting to desensitized conformation induces a large inward movement of loop 9 towards the channel lumen and an immobilization of loop 2, which is accompanied by substantial rearrangements of the intra- and intersubunit interface between the ECD and TMD. No appreciable proton-induced motions in the M2–M3 loop in the TMD were detected, demonstrating the conformation of this critical loop is similar in resting (closed, unliganded) and desensitized (closed, liganded) states. Proton-induced motions in GLIC were absent when the protein was in detergent micelles, indicating that lipids are required for functional gating transitions and suggesting that the detergents used for protein solubilization and crystallization may influence the conformations captured in the crystal structures. In general, residue positions and the proton-induced motions in functional GLIC protein embedded in lipid differ from those predicted based on the crystal structures of GLIC and ELIC obtained in detergent micelles, suggesting the GLIC crystal structure does not correspond to a desensitized conformation and that ELIC is not a suitable model for the structure of the M2–M3 loop of GLIC in the resting, closed state.

## Results

### Functional Characterization of Mutant GLIC Protein

To study proton-induced motions in loops forming the ECD/TMD interface of GLIC by EPR spectroscopy, we generated a cys-free GLIC mutant by replacing the lone native cysteine, C26, with alanine (Figure 1A,B). We then individually mutated K32 (loop 2), T157 (loop 9) and K247 and P249 (M2–M3 loop) to cysteine in the mutant C26A background (Figure 1A,B). We expressed wild-type and mutant proteins in *Xenopus laevis* oocytes and measured proton-induced currents using two-electrode voltage clamp (Figure S1B). All of the mutants formed functional channels with wild-type GLIC properties (pH_50_ = 5.2±0.1, Hill coefficient n_H_ = 1.6±0.1).

We also measured currents elicited by pH_50_ concentrations before and after reaction with the sulfhydryl-specific MTSL to determine if the wild-type cysteine (C26) and the introduced cysteines could be labeled by MTSL. For C26, K32C, T157C, and P249C, treatment with 1 µM MTSL for 2 min inhibited pH_50_ currents (30%–70%), demonstrating that the cysteines were accessible to modification with MTSL (Figure S1 and Table S1). The MTSL modification shifted the pH_50_ to more acidic values but did not alter maximal proton-activated currents (data not shown). For the mutants C26A and K247C, MTSL treatment had no effect on subsequent proton-activated currents. For K247C, treatment with the bulkier sulfhydryl-modifying reagent, MTSEA-biotin, inhibited proton-induced currents. To test whether MTSL modified K247C, we applied MTSL prior to MTSEA-biotin. MTSL blocked the ability of MTSEA-biotin to inhibit proton-induced currents, indicating that MTSL labels K247C but has no functional effect on channel activation.

**Figure 2:**
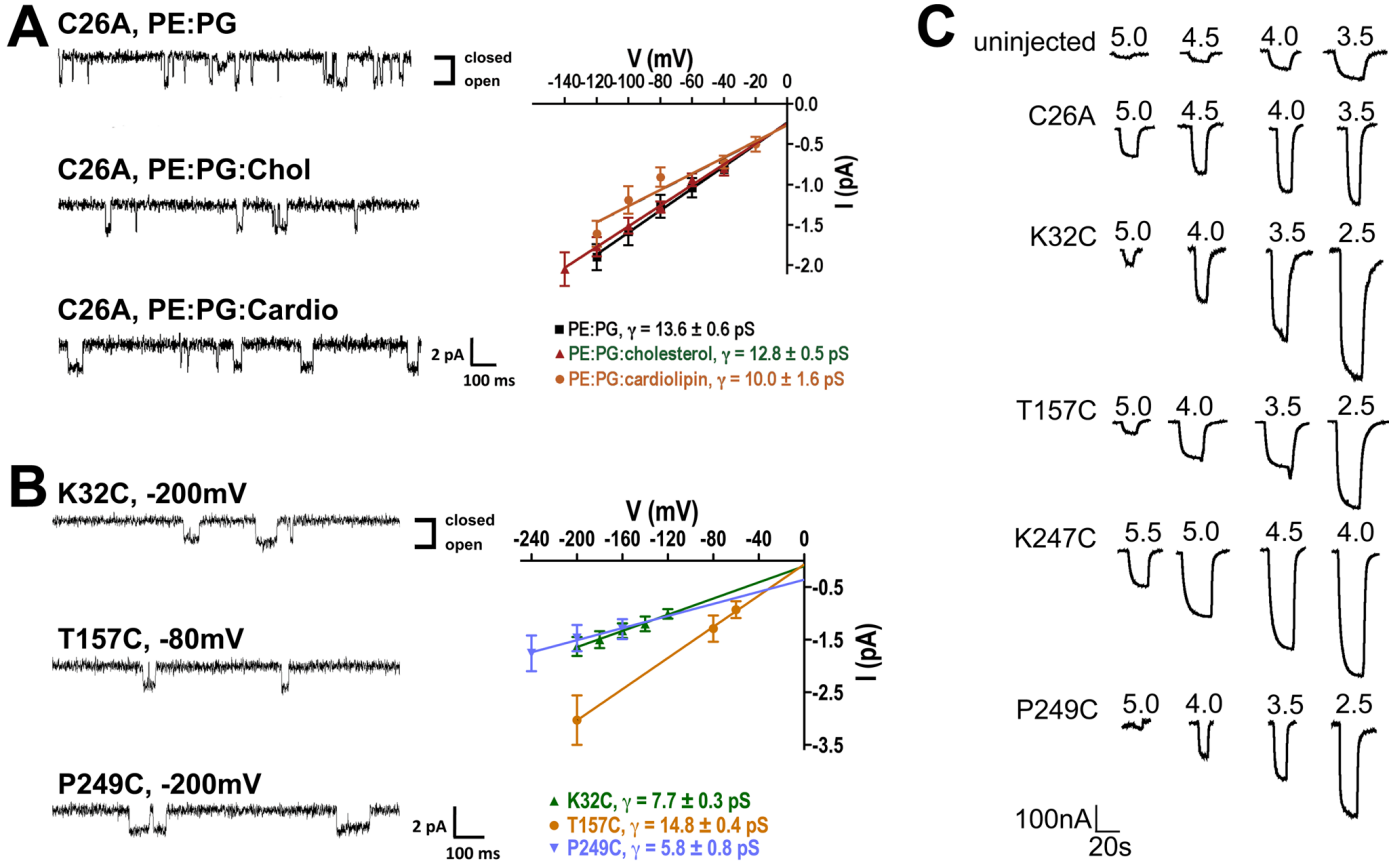
Purified GLIC reconstituted into liposomes is functional. (A) Single-channel currents of purified GLIC C26A mutant protein reconstituted into PE∶PG, PE∶PG∶cholesterol, and PE∶PG∶cardiolipin liposomes were recorded in planar lipid bilayers composed of the same lipids. Representative single-channel current traces (left), and current-voltage relationships with single-channel conductance values (right) are shown. Open-dwell times τ_o_ for the GLIC C26A mutant were 11.83±0.06 ms when reconstituted into PE∶PG liposomes, 10.15±0.08 ms into PE∶PG∶cholesterol liposomes, and 19.64±0.06 ms into PE∶PG∶cardiolipin liposomes, respectively. (B) Single-channel currents of purified GLIC mutants (K32C, T157C, and P249C) reconstituted into PE∶PG liposomes were recorded in planar lipid bilayers composed of the same lipids. Representative single-channel current traces (left) and current-voltage relationships with single-channel conductance values (right) are shown. (C) Currents induced by pH jumps from uninjected *Xenopus laevis* oocytes and ooctyes injected with purified, single cysteine MTSL-labeled GLIC protein reconstituted into PE∶PG liposomes (C26A, K32C, T157C, K247C, and P249C). Currents from GLIC-protein injected oocytes were significantly larger than those from uninjected oocytes.

We then expressed wild-type and mutant GLIC proteins in *E. coli*, purified the proteins in n-Dodecyl-β-D-maltoside (DDM), and labeled them with MTSL. To test whether the purified GLIC proteins were functional, we reconstituted mutant C26A into liposomes formed with 1-palmitoyl-2-oleoyl-sn-glycero-3-phosphoethanolamine (PE) and 1-palmitoyl-2-oleoyl-sn-glycero-3-phospho-(1′-rac-glycerol) (PG), and recorded single-channel currents in planar lipid bilayers also formed with PE∶PG (Figure 2A). PE and PG are major components of the inner cell membrane of most bacteria, and PE∶PG bilayers are good models for the bacterial membrane [24]. The purified C26A mutant reconstituted into PE∶PG liposomes produced proton-elicited single-channel currents with unitary conductance of 13.6±0.6 pS, which is comparable to the 8 pS value reported for wild-type GLIC in HEK293 cells [25].

We next tested whether the addition of cholesterol or cardiolipin along with PE and PG would affect GLIC single-channel properties. Cholesterol is essential for eukaryotic pLGIC function [26]–[30], and was recently shown to increase GLIC current desensitization rates [22]. Cardiolipin is an anionic lipid typically found in the bacterial cell membrane, and previous studies have reported that anionic lipids can modulate eukaryotic pLGIC function [29],[31]–[33]. We reconstituted mutant C26A into liposomes formed with PE∶PG∶cholesterol at a 3.4∶1.3∶1 molar ratio or PE∶PG∶cardiolipin at a 5.8∶2.3∶1 molar ratio, and recorded single-channel currents in planar lipid bilayers formed with the same lipids (Figure 2A). The single-channel conductance of C26A mutant GLIC was not altered by cholesterol (12.8±0.5 pS) or cardiolipin (10.0±1.6 pS). The open dwell time in the presence of cholesterol (10.15±0.08 ms, at −100 mV) was similar to that in PE∶PG (11.83±0.06 ms, at −100 mV), whereas in the presence of cardiolipin, it was slightly increased (19.64±0.06 ms, at −100 mV). Overall, the data demonstrate that purified GLIC C26A mutant protein reconstituted in PE∶PG liposomes is functional and that cholesterol and cardiolipin have little effect on the GLIC single channel properties measured.

We also confirmed that the purified reconstituted cysteine mutant GLIC proteins were functional. Single-channel currents recorded in PE∶PG bilayers for K32C, T157C, and P249C mutant protein (Figure 2B) had single channel conductances from 14.8±0.4 pS (T157C) to 5.8±0.8 pS (P249C), comparable to mutant C26A and wild-type GLIC [25]. In addition, we injected purified, reconstituted, MTSL-labeled protein directly into *Xenopus* oocytes to verify their functionality [34],[35]. We recorded significantly larger proton-dependent induced currents (approximately 300 nA–1 µA) from oocytes injected with mutant C26A, K32R1, T157R1, K247R1, and P249R1 as compared to noninjected oocytes (70 nA) (Figure 2C). Overall, the data demonstrate that the purified mutant GLIC proteins reconstituted into liposomes were functional and proton-sensitive.

### CW EPR Spectroscopy Reveals Proton-Induced Rearrangements

**Figure 3:**
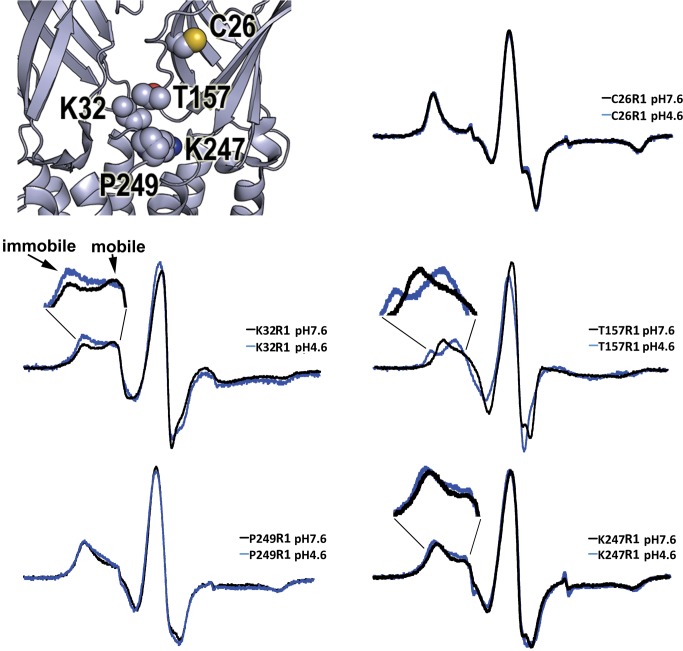
CW EPR spectra reveal proton-induced gating movements. Comparison of X-band CW EPR spectra of spin-labeled GLIC wild-type and mutant protein at pH 7.6 (black, closed state) and pH 4.6 (blue, desensitized state). Spectra were recorded at room temperature over 100 G. Pairs of data were recorded on the same spectrometers and under identical conditions. Immobile and mobile components in the low-field region of the K32R1 spectrum are indicated by arrows. The low-field regions of the K32R1, T157R1, and K247R1 spectra are enlarged to highlight the proton-induced changes. (Top left) Close-up view of GLIC crystal structure with spin-labeled positions C26, K32, T157, K247, and P249 shown in space-fill.

We initially recorded the CW spectra of MTSL-labeled wild-type GLIC (C26R1), at pH 7.6 and pH 4.6 (Figure 3). The shape of the CW spectrum reflects the mobility of the R1 side chain, which ultimately depends on packing of its surroundings. Therefore, proton-induced changes in the CW spectrum reflect structural rearrangements that alter the local environment around R1 (i.e., neighboring side chain motions and backbone flexibility). The CW EPR measurements were collected at room temperature over an hour (steady-state conditions). Based on our proton-concentration response curves, at pH 7.6, the channels will predominantly be in an unliganded, resting conformation. At pH 4.6, the channels will predominantly be in a desensitized conformation. At both pH values, the CW spectra of C26R1 showed the spin labels were largely immobile (Figure 3), indicating a tightly packed environment near the C26R1 side-chain, consistent with its buried location on β-strand 1. Switching to pH 4.6 had no effect on the shape of the C26R1 CW spectrum, indicating that probe mobility did not change, which suggests the local environment near the probe is the same or it rearranged into an equally packed conformation. The CW spectrum of an MTSL-treated C26A mutant showed virtually no signal (Figure S2), demonstrating the absence of spin-labeled protein contaminants.

**Figure 4:**
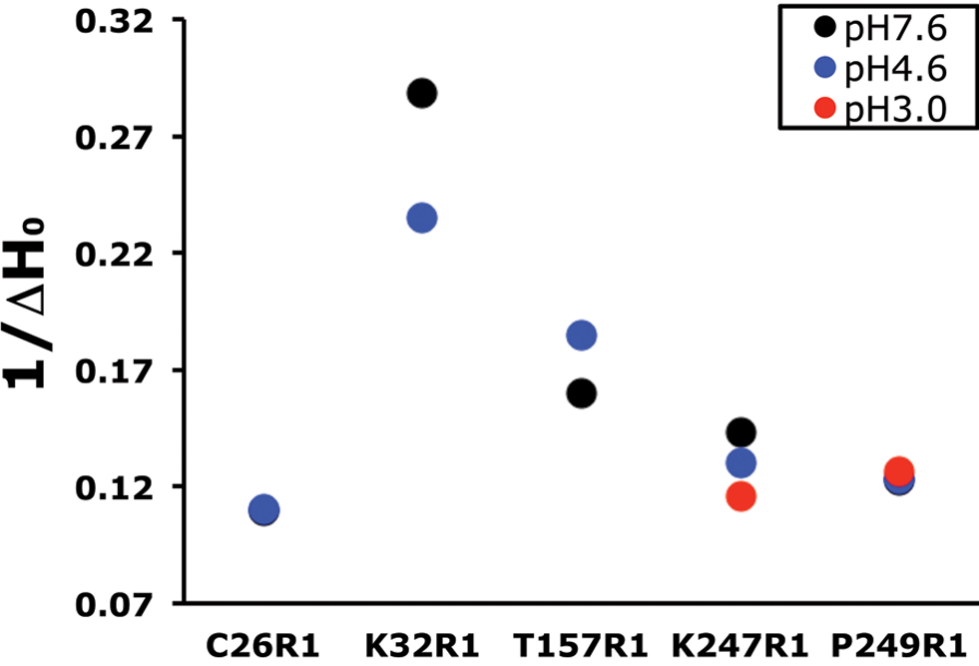
Proton-induced changes in spin probe mobility, ΔH_0_^−1^. For each GLIC mutant (C26R1, K32R1, T157R1, K247R1, and P249R1), the inverse width of the central line of the CW spectra, ΔH_0_^−1^, is plotted at pH 7.6 (black), pH 4.6 (blue), and pH 3.0 (red, K247R1 and P249R1 only). An increase in ΔH_0_^−1^ reflects increased R1 mobility, whereas a decrease reflects decreased mobility.

To detect proton-induced conformational rearrangements in loop 2, loop 9, and the M2–M3 loop, we recorded the CW spectra of the MTSL-labeled GLIC mutants K32R1, T157R1, K247R1, and P249R1 at pH 7.6 and pH 4.6 (Figure 3). In the ECD, the CW spectra of K32R1, located in loop 2, showed two distinct EPR spectral components, mobile and immobile (indicated by arrows in Figure 3), likely associated with two alternative rotameric spin-label conformations [36]. At pH 7.6 (closed, resting state), a greater proportion of the spin probes were in a mobile conformation, whereas at pH 4.6 (desensitized) a greater proportion were immobile, indicating a proton-induced structural rearrangement occurred that resulted in a more densely packed environment near the spin probe. Proton-induced changes were also detected in the CW spectra of T157R1 (Figure 3), located in loop 9. The low field regions of the spectra were entirely different at the two pH values, indicating a completely new motional environment, with the spin probes predominantly immobile at pH 7.6 (resting) and mostly mobile at pH 4.6 (desensitized). For both K32R1 and T157R1, switching to pH 4.6 did not result in significant spectral broadening or changes in center resonance line amplitude, indicating that the observed differences reflect changes in probe mobility and not intersubunit dipolar spin–spin interactions. Changes in spin probe mobility are plotted in Figure 4 and were calculated by measuring the inverse width of the central line, ΔH_0_^−1^: an increase in ΔH_0_^−1^ indicates an increase in motion, whereas a decrease in ΔH_0_^−1^ indicates a decrease in motion [16],[17],[37].

**Figure 5:**
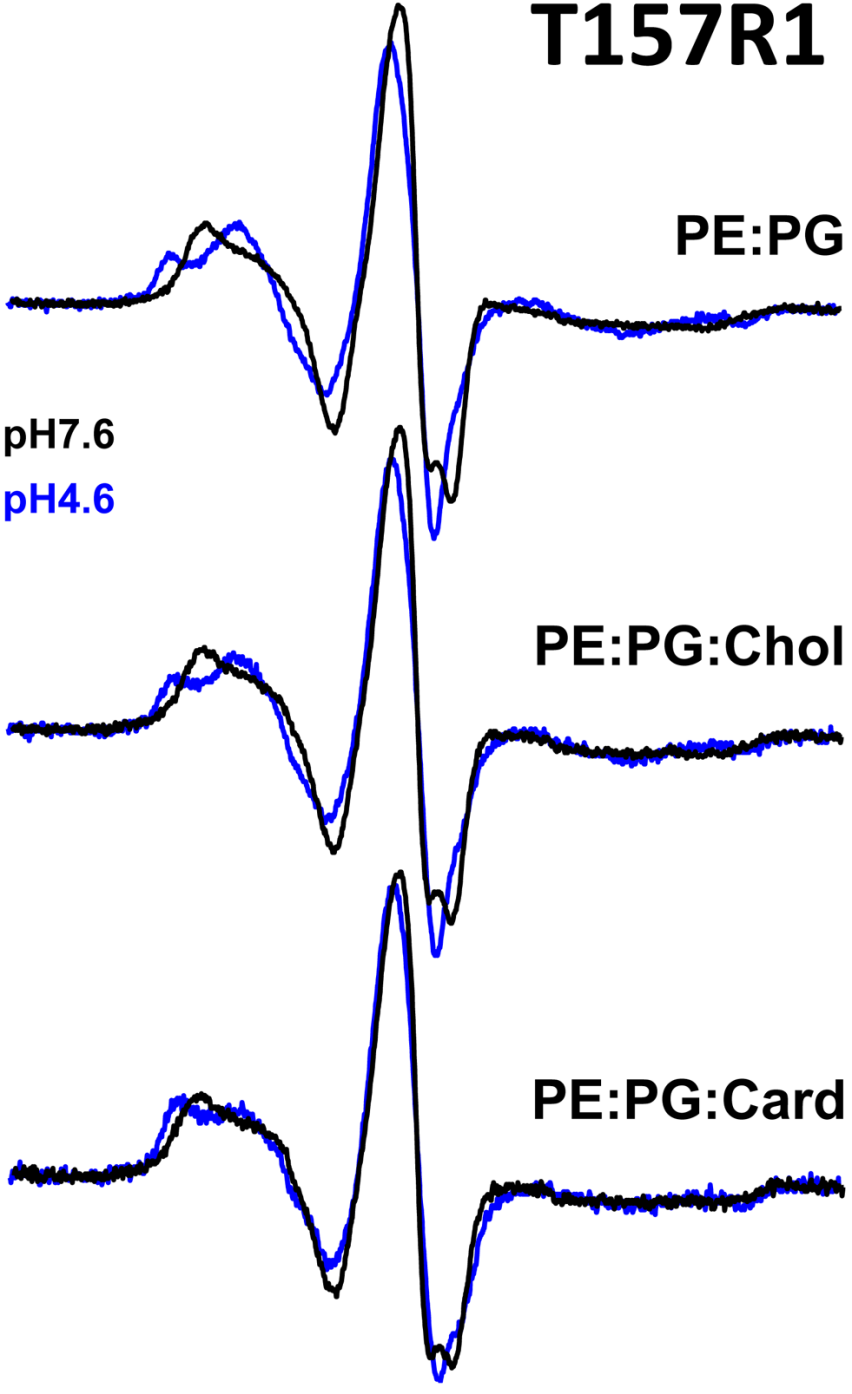
Effects of lipids on proton-induced gating motions. X-band CW EPR spectra of T157R1 reconstituted into PE∶PG (top), PE∶PG∶cholesterol (middle), and PE∶PG∶cardiolipin (bottom) liposomes at pH 7.6 (black, resting state) and pH 4.6 (blue, desensitized). Proton-induced changes in T157R1 mobility in the presence of cholesterol were indistinguishable from those of T157R1 reconstituted in PE∶PG, whereas cardiolipin hindered gating-induced changes in T157R1 mobility.

We also collected CW spectra of T157R1 reconstituted in PE∶PG∶cholesterol and in PE∶PG∶cardiolipin at pH 7.6 and pH 4.6 (Figure 5) to test the effects of lipids on proton-induced motions in GLIC. In the presence of cholesterol, the CW spectra were essentially indistinguishable from the spectra of T157R1 reconstituted in PE∶PG, indicating that cholesterol had no effect on the proton-induced structural rearrangements near loop 9. In the presence of cardiolipin, there was a marked decrease in the population of spin probes that switched to the more mobile conformation at pH 4.6 (Figure 5), suggesting that cardiolipin hinders proton-induced motions near loop 9.

In the TMD, the CW spectra of K247R1 and P249R1, located in the M2–M3 loop, also revealed the spin labels were motionally restricted, indicating a sterically packed environment (Figure 3). For K247R1, we observed an additional slight decrease in mobility upon switching to pH 4.6 and no changes in mobility for P249R1. The lack of significant proton-induced changes in probe mobility was unexpected, since motions in the M2–M3 loop have been suggested to play an important role in coupling agonist binding to channel gating [7],[38]–[40]. To ensure that we were maximally activating the MTSL-labeled GLIC protein, we also collected CW spectra at pH 3.0 (Figure S3). Upon changing the pH to 3.0, probe mobility for K247R1 decreased slightly more, whereas no changes were seen for P249R1, as judged by the inverse central line width ΔH_0_^−1^ (Figure 4). In general, no significant changes in the overall line shape of the CW spectra at pH 3.0 compared to pH 4.6 or pH 7.6 were observed (Figure S3).

### Measuring Distances and Proton-Induced Distance Changes Using DEER Spectroscopy

**Figure 6:**
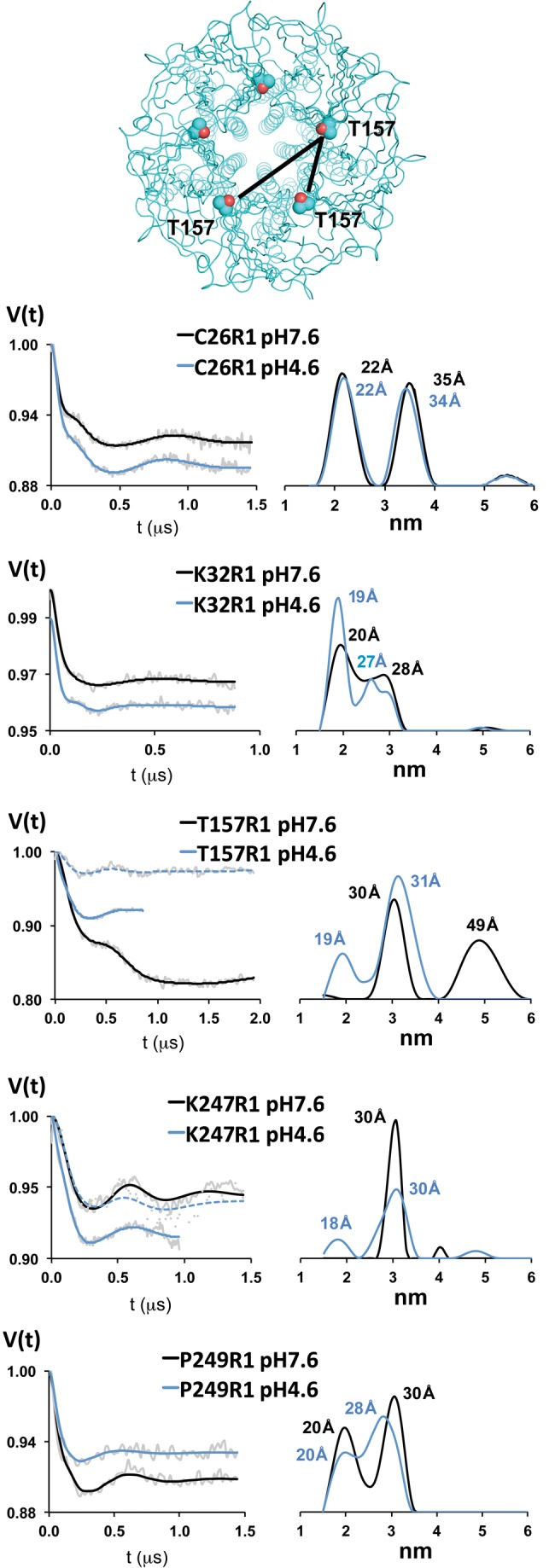
Proton-induced distance changes revealed by DEER spectroscopy. (Top panel) Top-down view of GLIC crystal structure shown in ribbon representation with T157 in spacefill, black lines depict distances between adjacent and nonadjacent residues. (Left panels) Background subtracted Q-band DEER-refocused echo intensity (grey lines) is plotted versus evolution time for each spin-labeled position at pH 7.6 (resting state) and pH 4.6 (desensitized state) and fit using model-free Tikhonov regularization (pH 7.6, black lines; pH 4.6, blue lines). Pairs of data were recorded on the same spectrometers and under identical conditions. (Right panels) The corresponding interspin distance distributions are plotted at pH 7.6 (black) and pH 4.6 (blue) with the mean distances for each peak labeled. For T157R1 and K247R1 samples at pH 4.6, we also collected data out to the same dipolar evolution times as the pH 7.6 samples (dotted blue lines).

Using DEER spectroscopy, distances in the range of 18 to 60 Å between paramagnetic centers in a membrane protein can be measured [41]–[43]. Because GLIC is a homopentamer, two distances are expected at each labeled position: one between spin probes on adjacent subunits, another between probes on nonadjacent subunits (Figure 6, Figure S4A) with a theoretical nonadjacent and adjacent distance ratio of 1.6 expected. We measured the distances between probes in GLIC at pH 7.6, which stabilizes the closed state, and at pH 4.6, which favors desensitized states, to test if, and how far, the loops at the ECD/TMD gating interface (e.g., K32R1, T157R1, K247R1, and P249R1) move during proton-dependent gating transitions. Currently, a high-resolution structure of GLIC is only available in an apparently open channel conformation, and little is known about the process of desensitization at the structural level. While there are uncertainties in assigning functional states to the ELIC and GLIC crystal structures, comparing ELIC (PDB entry 2VL0) and GLIC (PDB entry 3EHZ) solved in apparently closed and open channel conformations, respectively [6],[7],[10], suggests that loops 2 and 9 move inward toward the channel lumen (∼1.7 Å for K32 relative to ELIC's *L29*, and ∼5 Å for T157 relative to ELIC's *D158*), whereas the M2–M3 loop moves ∼6 Å outward away from the channel lumen (K247 relative to ELIC's *R254* and P249 relative to ELIC's *P256*) with channel activation (Figure 1C,D; Table 1; see Materials and Methods and Figure S4B for displacement calculations). By comparing our experimental DEER distances obtained from functional protein in lipids to those predicted from the crystal structures, we can begin to assess the conformational states to which these structures correspond.

**Table 1:** Summary of distances.

	pH 7.6				pH 4.6				
Residue	Short (Å)	Width (Å)	Long (Å)	Width (Å)	Short (Å)	Width (Å)	Long (Å)	Width (Å)	δ (Å)
**C26R1**	22	5	35	5	22	5	34	5	0
*I23ELIC/C26GLIC*	*24.7*		*40*		*24.2*		*39.1*		*0*
**K32R1**	20	6	28	7	19	4	27	7	0.7, inward
*L29ELIC/K32GLIC*	*16.1*		*26*		*14*		*22.7*		*1.7, inward*
**T157R1**	30	5	49	9	19	6	31	7	9.2, inward
*D158ELIC/T157GLIC*	*27.6*		*44.7*		*21.7*		*35.1*		*5, inward*
**K247R1**			30	3	18	5	30	6	0
*R254ELIC/K247GLIC*	*13.8*		*22.3*		*20.8*		*33.7*		*6, outward*
**P249R1**	20	6	30	5	20	7	28	7	0
*P256ELIC/P249GLIC*	*23.1*		*37.4*		*30.1*		*48.7*		*6, outward*

In roman type, adjacent (short) and nonadjacent (long) interspin distances and corresponding peak widths measured by DEER spectroscopy at pH 7.6 and pH 4.6 for GLIC mutants reconstituted into PE∶PG liposomes. In italics, adjacent (short) and nonadjacent (long) interresidue C_β_–C_β_ distances for C26, K32, T157, K247, and P249 obtained from GLIC (PDB entry 3EHZ, pH 4.6 distances), and for aligned residues I23, L29, D158, R254, and P256 in ELIC (PDB entry 2VL0, pH 7.6 distances). Last column shows the calculated intrasubunit displacement δ values (see Materials and Methods and Figure S4).

We initially examined C26R1, which is located on β-strand 1 in the ECD, for intersubunit distances by DEER spectroscopy. Figure 6 shows the background subtracted dipolar evolution fit using Tikhonov regularization, a model-free approach. The interspin DEER-derived distances were 22 Å and 35 Å at pH 7.6 (adjacent and nonadjacent subunits, respectively), and 22 Å and 34 Å at pH 4.6 (Figure 6, Table 1). Similar distance distributions were obtained when we fit the data using 2-Gaussian or 2 Rice_3D_ model-based approaches. The nonadjacent∶adjacent distance ratios were 1.6, in excellent agreement with the theoretical value for a homopentameric labeled protein. The DEER-derived interspin distances were slightly shorter than the C_β_–C_β_ distances (Table 1) measured in the crystal structures of ELIC and GLIC. The absence of detectable pH-induced distance changes indicates either a lack of motion or a concerted rigid-body motion for the ECD β-cores.

For K32R1, in loop 2, the experimental distances were 20 Å and 28 Å at pH 7.6 (adjacent and nonadjacent subunits, respectively), and 19 Å and 27 Å at pH 4.6 (Figure 6, Table 1). The nonadjacent∶adjacent distance ratios were 1.4, which are smaller than the 1.6 theoretical value, suggesting that the probe locations were not perfectly symmetrical. The small (less than 1 Å) proton-dependent change in the interprobe center distances suggests that there is little to no proton-induced displacement of loop 2.

For T157R1, in loop 9, the interspin DEER-derived distances were 30 Å and 49 Å at pH 7.6 (adjacent and nonadjacent subunits, respectively), and 19 Å and 31 Å at pH 4.6 (Figure 6). The nonadjacent∶adjacent distance ratios were 1.6, in excellent agreement with the theoretical value for a homopentameric labeled protein. At pH 4.6, we collected data out to a shorter dipolar evolution time (solid blue line) to increase the quality of the data. When we collected data out to the same evolution time as the pH 7.6 sample (dotted blue line), intersubunit distances longer than 31 Å were not observed. Upon switching to pH 4.6, the interprobe distance changed more than 17 Å for the nonadjacent distance, indicating that the probe attached to loop 9 undergoes a large proton-induced inward movement toward the channel lumen, with a displacement of 9.2 Å (Figure S4).

**Figure 7:**
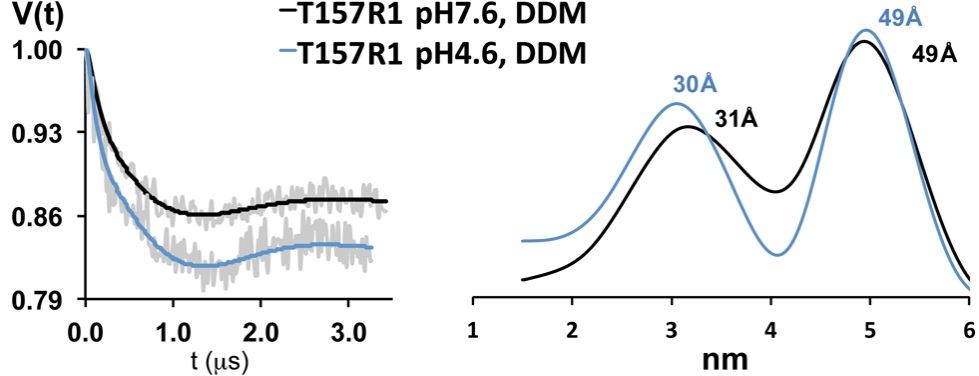
Detergent prevents proton-induced GLIC gating motions. (Right panel) Interspin distance distributions from model-free Tikhonov fits of X-band DEER data from GLIC T157R1 purified in detergent (DDM) micelles at pH 7.6 (black) and pH 4.6 (blue). (Left panel) The background-corrected dipolar evolution data at pH 7.6 and pH 4.6 (grey lines) and the Tikhonov fits (pH 7.6, black lines; pH 4.6, blue lines). The interspin DEER-derived distances are similar at both pH values, indicating that detergent-solubilized GLIC does not undergo proton-mediated gating motions.

Since T157R1 is a good reporter of proton-induced conformational motions, we examined the ability of GLIC to undergo these rearrangements in detergent micelles. For T157R1 in DDM micelles, the interspin DEER-derived distances were 31 Å and 49 Å at pH 7.6 (adjacent and nonadjacent subunits, respectively), and 30 Å and 49 Å at pH 4.6 (Figure 7). The distances were nearly identical at both pH values and matched the distances obtained from GLIC reconstituted into PE∶PG liposomes at pH 7.6—that is, in the resting, closed channel state (Figure 6 and Table 1). The data suggest that DDM inhibits proton-induced motions in GLIC and locks GLIC in a conformation resembling the resting state.

For K247R1, in the M2–M3 loop, at pH 7.6 only one interspin distance of 30 Å was obtained using Tikhonov regularization (Figure 6). These data were also fit using 2-Gaussians and 2 Rice_3D_ model-based approaches, which also resulted in only one distance (i.e., two distances of the same value resulted). The interspin distance does not correlate with either of the C_β_–C_β_ distances in the ELIC crystal structure (13.8 Å adjacent, 22.3 Å nonadjacent) but is comparable to the nonadjacent C_β_–C_β_ distance in GLIC (33.7 Å). At pH 4.6, we detected two distances of 18 Å and 30 Å (adjacent and nonadjacent subunits, respectively) similar to C_β_–C_β_ distances measured in the GLIC crystal structure (Table 1). The apparent absence of any proton-induced change in the nonadjacent 30 Å distance suggests that K247R1 occupies the same location at both pH values, which is consistent with the very modest changes we observed in the CW EPR spectra upon switching to pH 4.6 (Figure 3). We cannot, however, rule out the possibility that the interspin 30 Å distance measured at pH 7.6 is between adjacent subunits. Given the short phase memory time for this position (T_m_ = 0.6 µs), a nonadjacent distance of approximately 49 Å (expected for a 30 Å adjacent distance) is beyond the range that can be reliably measured [41]–[43].

For P249R1, also in the M2–M3 loop, the interspin distances were 20 and 30 Å (adjacent and nonadjacent subunits, respectively) at pH 7.6, and 20 Å and 28 Å at pH 4.6 (Figure 6), indicating little to no proton-induced changes in distances, consistent with the lack of proton-elicited changes seen in the CW EPR spectra (Figure 3). Overall, the data suggest that the M2–M3 loop is in a similar position in both the resting and desensitized GLIC conformational states. This result is in contrast to its approximately 6 Å outward displacement away from the channel lumen predicted by comparing the crystal structures of ELIC and GLIC [7].

### Estimating Distances by Computer Modeling

We also used computer modeling to evaluate how well the ELIC and GLIC crystal structures predict our experimental DEER data. We built a homology model of GLIC based on the ELIC crystal structure and used the PRONOX program to estimate the distances between spin labels in the GLIC model (Table S2). The computed distances were then compared to our experimental DEER distances. Using standard conditions in the program, no distances were computed for C26R1, T157R1, and P249R1 due to clashes (i.e., MTSL did not fit using favored rotamer conformations), and the average interspin distances computed for K32R1 and K247R1 were shorter than our experimental data. When we relaxed conditions and allowed additional MTSL rotamer conformations, the program still could not compute distances for C26R1 and P249R1 and the distance for T157R1 was shorter than our experimental data. In general, the modeling suggests the ELIC structure obtained in detergent micelles, at least for these positions, is not a good model for the resting state of GLIC embedded in lipids. We also used the PRONOX program to estimate the distances between spin labels using the crystal structure of GLIC (PDB entry 3EAM) as the input (Table S2). Using standard or relaxed conditions, no distances were computed for C26R1. For T157R1 and K247R1, the estimated distances were similar to our experimental DEER distances obtained at pH 4.6. The computed distances for K32R1 and P249R1 were much shorter than the experimental DEER distances at pH 4.6, suggesting the GLIC crystal structure, at least at these positions, does not correspond to a desensitized conformation.

## Discussion

The structural rearrangements underlying how pLGICs transition between closed, open, and desensitized states are still unclear. While high-resolution crystal structures of pLGICs in apparently closed and open channel conformations [6],[7],[9],[10] have provided insights into possible activation mechanisms, whether these static protein structures, solved in detergent micelles, accurately capture the conformational gating transitions that a functional pLGIC undergoes when embedded in a lipid bilayer is unknown. Using SDSL EPR spectroscopy and functional GLIC channels reconstituted in liposomes, we measured protein motions associated with GLIC gating under conditions that promote conformational transitions from closed to desensitized states. We focused on the loops forming the interface between the ECD and the TMD, specifically loops 2 and 9 in the ECD and the M2–M3 loop in the TMD, which previous studies have shown to be important for coupling agonist binding to channel gating [39],[40],[44],[45]. Comparing the crystal structures of ELIC and GLIC suggests that these loops undergo structural rearrangements during activation, with loops 2 and 9 moving inward toward the channel lumen, whereas the M2–M3 loop moves outward (Figure 1C,D). Whether and if these loops move during desensitization is unknown.

Here, we show that proton-dependent GLIC channel gating transitions into a desensitized state induces substantial rearrangements of the intra- and intersubunit interface between the ECD and TMD. The biggest change occurred in loop 9, where T157R1 underwent a large (∼9.2 Å) proton-induced inward movement toward the channel lumen (Figure 6). The displacement was accompanied by concurrent rearrangements in its surrounding tertiary contacts, with the CW spectra (Figure 3) revealing a densely packed environment in the resting state (pH 7.6) that becomes less packed in the desensitized state (pH 4.6). The DEER-derived interspin distances for T157R1 at pH 7.6 are longer than the C_β_–C_β_ distances in the ELIC crystal structure and the intersubunit distances estimated computationally, whereas the DEER distances at pH 4.6 are shorter than the C_β_–C_β_ distances in the GLIC crystal structure and the distances estimated computationally (Table 1, Table S2). Thus, the resulting proton-mediated 9.2 Å inward displacement of loop 9 measured by DEER spectroscopy is larger than the predicted motion based on comparing the ELIC and GLIC crystal structures. Since the residues in loop 9 in ELIC and GLIC differ substantially [7],[10], it is not surprising that the magnitude of the DEER-derived displacement measured in a single protein, GLIC, does not match the displacement calculated by comparing the structures of two different proteins. Also, the loop 9 displacement measured by DEER spectroscopy in a functional protein reconstituted in liposomes may differ from the motion observed when the protein is constrained in a crystal lattice in detergent. In support of this latter possibility, our DEER data demonstrate that the proton-mediated motion of T157R1 is lost in DDM micelles compared to T157R1 reconstituted in PE∶PG liposomes (Figures 6 and 7). This finding is consistent with the loss of channel function observed for eukaryotic nicotinic acetylcholine receptors solubilized in DDM [46] and supports the idea that detergent-solubilization of membrane proteins can affect structural dynamics and result in conformational ambiguity of the crystal structures solved in the presence of detergents (reviewed in [13]).

Our DEER spectroscopy experiments measured the intersubunit distances of T157R1 in GLIC in a resting, closed state (pH 7.6) and in a desensitized, closed state (pH 4.6). Currently, our experiments cannot distinguish whether loop 9 moves in the open state and remains displaced during desensitization or whether its movement occurs specifically in the desensitized state. Nonetheless, the data directly demonstrate that a large inward motion of loop 9 occurs during GLIC gating transitions, which results in substantial rearrangements of the intersubunit interface. We predict that a similar inward motion of loop 9 in eukaryotic pLGICs occurs during agonist-mediated channel gating transitions. By measuring changes in cysteine accessibility, disulfide bond formation, and attached fluorophore emissions, agonist-induced local rearrangements near loop 9 have been detected in nicotinic acetylcholine receptors [47],[48], GABA_A_ receptors [49]–[52], glycine receptors [53], and serotonin-type 3 receptors [54].

Proton-mediated structural rearrangements in the local protein environment near K32R1 in loop 2 were also observed. CW EPR spectroscopy (Figure 3) revealed K32R1 is in a more densely packed environment in the desensitized state (pH 4.6) compared to the resting state (pH 7.6), suggesting that during channel activation to desensitization loop 2 becomes less mobile. Since our DEER measurements at pH 7.6 and 4.6 demonstrate loop 2 undergoes minimal proton-induced displacement (Figure 6), the decrease in K32R1 mobility likely arises primarily from an increase in its surrounding tertiary interactions. Residues in loop 7, loop 9 (adjacent subunit), M2–M3 loop, and the pre-M1 are in close proximity to loop 2, and functional studies in eukaryotic pLGICs have shown that a network of electrostatic and hydrophobic interactions between these regions and loop 2 play a role in coupling binding to gating [45],[55]. Our data suggest that increases in these interactions and a resulting immobilization of loop 2 accompany GLIC channel gating transitions into the desensitized state.

Proton-induced conformational rearrangements near the M2–M3 loop in the TMD were minimal, at least for the two positions we examined, K247R1 and P249R1. In the resting closed channel state (pH 7.6), the EPR spectra (Figure 3) showed the spin-probes at both positions were motionally restricted, reflecting a sterically packed environment near the probes. Upon switching to pH 4.6 or 3.0 (desensitized state), only a modest decrease in K247R1 mobility was observed, whereas no change in P249R1 mobility was seen (Figures 3 and 4). Moreover, our DEER data indicate that there were essentially no proton-mediated changes in intersubunit distances between probes at these positions (Figure 6). Overall, our data suggest the M2–M3 loop does not undergo significant movement.

Based on the crystal structures of ELIC and GLIC, the M2–M3 loop is predicted to move ∼6 Å (C_β_–C_β_) outward away from the channel lumen during activation (Figure 1C and Table 1). One possible explanation for the difference between our data and the structure-based predictions is that in the desensitized state, which we are preferentially monitoring at pH 4.6, the M2–M3 loop adopts a conformation that resembles its conformation in the resting state. Consistent with this idea, photolabeling of a residue in the M2–M3 loop of the nicotinic acetylcholine receptor delta subunit is state-dependent with robust labeling seen only in open and fast desensitized states and little to no labeling in the resting and slow desensitized states [56]. In a cysteine accessibility study, modification of cysteines introduced into the GLIC M2 helix and the M2–M3 loop were faster at pH 5.0 than pH 7.5, indicating a proton-mediated increase in accessibility [12]. Again, a possible explanation for the difference between their data and ours is that we are monitoring desensitized states at pH 4.6, whereas in their study the channels are submaximally activated at pH 5.0 and in a mixture of open and possibly resting and desensitized conformations. Overall, our SDSL EPR data suggest that the M2–M3 loop is in a relatively packed environment in the resting state that is relatively unchanged in the desensitized state. A recent work [23] suggests residues in the middle of M2 move inward to occlude the channel during desensitization, whereas residues in the extracellular end of M2 remain displaced outward. One might expect then that the M2–M3 loop, which is attached to M2, would remain displaced outward. Our data suggest that this is not the case.

Whether the GLIC M2–M3 loop moves substantially during proton-induced channel opening and then moves back to a position similar to that adopted in the resting state during desensitization is unclear. The motions inferred from static crystal structures of two different proteins in uncertain functional conformations might not accurately reflect gating motions of a functional protein embedded in a lipid membrane. The intersubunit distances we measured at pH 7.6 in the resting state for both K247R1 and P249R1 are substantially different from the C_β_–C_β_ distances in the crystal structure of ELIC for the structurally aligned residues R254 and P256 and from the intersubunit distances that we estimated computationally, suggesting that the ELIC structure is not a good model for the conformation of GLIC's M2–M3 loop in the resting state. In the recently crystallized locally closed GLIC structures, the three conformations of the M2–M3 loop are different than the M2–M3 loop conformation in ELIC [57]. In addition, comparing one of the locally closed channel GLIC structures (LC2 conformation, PDB entry 3TLS) with GLIC in an apparently open channel state (PDB entry 3EAM) suggests channel closing/opening can occur without significant rearrangements of the M2–M3 loop.

MTSL labeling and/or detergent solubilization and membrane-reconstitution could lock the receptor in a nonactivatable “uncoupled” state, which abolishes its ability to undergo conformational transitions in response to pH changes. Previous work on nicotinic acetylcholine receptors have shown that lipid composition and choice of detergent are critically important for maintaining optimal receptor functionality [32],[33],[46],[58]. Since we record robust proton-elicited currents in bilayers with our purified PE∶PG lipid reconstituted GLIC protein, and when we directly inject purified MTSL-labeled lipid reconstituted protein into oocytes (Figure 2), these possibilities seem unlikely. Moreover, in a recent study, the ability of GLIC to undergo proton-dependent gating transitions was maintained following its reconstitution in a variety of different lipids [59], consistent with our findings.

Both cholesterol and anionic lipids are well-known modulators of pLGIC function [60], and it was recently shown that cholesterol modulates GLIC gating kinetics, speeding its desensitization [22]. Our data using CW EPR spectroscopy showed that cholesterol, when added to PE∶PG liposomes, had no effect on the proton-induced structural rearrangements near loop 9 (Figure 5). In addition, cholesterol had little effect on GLIC single channel conductance or open-dwell time. On the other hand, adding cardiolipin to PE∶PG liposomes reduced probe mobility at pH 4.6 compared to PE∶PG alone or PE∶PG∶cholesterol, suggesting that cardiolipin inhibits proton-induced motions near loop 9 (Figure 5). In a recent report, GLIC protein appeared slightly more rigid when reconstituted into membranes formed by *E. coli* lipids (i.e., a PE∶PG∶cardiolipin mixture) as compared to reconstitution in asolectin or phosphocholine [59], consistent with our finding that cardiolipin decreases GLIC mobility.

In summary, using SDSL EPR spectroscopy, we present new information about the structural changes associated with ligand-induced gating motions in the prokaryotic pLGIC GLIC using a functional protein reconstituted into a native-like lipid environment. We provide direct experimental evidence that structural rearrangements of the intra- and intersubunit interface between the ECD and TMD accompany pLGIC gating transitions from closed to desensitized states. Specifically, in the ECD, proton-induced gating transitions from closed to desensitized states decrease local side-chain interactions with loop 9, which increases loop 9 mobility and results in a large inward movement of loop 9, whereas loop 2 becomes more immobilized. These data suggest that desensitization not only involves structural changes in the M2 channel helix to block ion conduction [23] but also entails motions in the ECD that likely change the network of interactions between residues in loop 2, loop 7, loop 9, preM1, and the M2–M3 linker. In the resting state, the M2–M3 loop in the TMD domain is relatively immobile and in a packed environment, and remains in nearly the same position in the desensitized state. The position of P249R1 in the M2–M3 loop in the desensitized state is substantially different than that observed in the GLIC apparently open channel structure, suggesting the crystal structure is not in a desensitized conformation. Currently, a resting, closed channel state structure of GLIC is not available. Our DEER data provide a first glimpse of the positions of GLIC residues in the resting state and suggest that the ELIC structure is not a good model for the resting state. These findings advance our understanding of the molecular mechanisms underlying pLGIC gating.

## Materials and Methods

### Cloning and Mutagenesis

The DNA sequence encoding GLIC (residues 44–359) was extracted by PCR amplification from *G. violaceus* cells (ATCC), and subcloned in vectors pUNIV [61] for two-electrode voltage clamp experiments, and pET-26b (Novagen) for expression in *E. coli*. GLIC DNA sequence was preceded in pUNIV by the DNA sequence encoding the signal peptide of the GABA_A_ receptor β2 subunit to promote cell surface expression. pET-26b incorporates an N-terminal *pelB* signal sequence for potential periplasmic localization. In addition, DNA sequence for maltose-binding protein (MBP) followed by a ∼20 amino acid linker containing a consensus sequence for thrombin cleavage was cloned following the *pelB* signal and N-terminal to GLIC. GLIC mutants were created using the QuikChange site-directed mutagenesis kit (Stratagene). Mutations were confirmed by DNA sequencing.

### Two-Electrode Voltage Clamp Recordings in *Xenopus laevis* Oocytes

Capped cRNAs encoding WT and mutant GLIC were transcribed in vitro using the mMessage mMachine T7 kit (Ambion). Single *X. laevis* oocytes were injected with 27 nL of cRNA (50–100 ng/µL/subunit). Injected oocytes were incubated at 16°C in ND96 (5 mM HEPES pH 7.4, 96 mM NaCl, 2 mM KCl, 1 mM MgCl_2_, 1.8 mM CaCl_2_) supplemented with 100 µg/ml of gentamycin and 100 µg/mL of bovine serum albumin for 2–5 d before use for electrophysiological recordings. Oocytes were perfused continuously with ND96 at pH 7.4 at a flow rate of 5 mL/min, while being held under two-electrode voltage clamp at −60 mV in a bath volume of 200 µL. Borosilicate glass electrodes (Warner Instruments) used for recordings were filled with 3 M KCl and had resistances of 0.4 to 1.0 MΩ. Electrophysiological data were collected using Oocyte Clamp OC-725C (Warner Instruments) interfaced to a computer with an ITC-16 A/D device (Instrutech) and were recorded using the Whole Cell Program, version 4.0.9 (kindly provided by J. Dempster, University of Strathclyde, Glasgow, UK). Proton-induced currents were measured by perfusing ND96 buffered at pH 6.5–3.8. For pH 5.0–3.8 HEPES was replaced with 5 mM Na Citrate as the buffering agent. For pH 6.5–6.0 5 mM MES was used as the buffering agent. GraphPad Prism 4 was used for data analysis and fitting. pH response data were fit to the equation I = I_max_/(1+10^(pH-pH50)*n^), where I is the peak response at a given pH, I_max_ is the maximum amplitude of current, pH_50_ is the pH inducing half maximal response, and n is the Hill coefficient. The functional effect of modifying substituted cysteines with 1-oxyl-2,2,5,5-tetramethyl-3-pyrroline-3-methyl methanethiosulfonate spin label (MTSL) was evaluated in oocytes using two-electrode voltage clamp. Proton-induced currents were measured at pH 5.0 until peak current amplitudes varied by <5%. Oocytes were then treated with 1 µM MTSL at pH 7.4 for 2 min, washed for 5 min, and proton-induced currents were measured again at pH 5.0. Extent of modification was quantified as (1−I_after MTSL_/I_before MTSL_)*100%. WT and C26A were incubated with 100 µM MTSL.

### Protein Expression and Purification

*E. coli* BL21(DE3) strain cells (Invitrogen) were transformed with the pET-26b vector encoding the GLIC constructs. Cells were cultured in LB medium at 37°C to OD_600_∼1.0–1.4, and then expression was induced overnight at 20°C with 0.2 mM isopropyl β-D-1-thiogalactopyranoside (IPTG). Cells were harvested and lysed with an EmulsiFlex C-5 homogenizer (Avestin) in 20 mM Tris-HCl pH 7.6, 150 mM NaCl (buffer B1) supplemented with 1 mM PMSF, 2 µM pepstatin-A, and 2 µg/mL leupeptin as protease inhibitors. The lysate was cleared by centrifugation at 18,000 rpm for 30 min at 4°C, and then the pellet was resuspended in B1 with 2% n-dodecyl-β-D-maltoside (DDM, Anatrace) and gently agitated overnight at 4°C for protein extraction from cell membranes. Solubilized pellet was cleared by ultracentrifugation at 45,000 rpm with a 50.2 Ti rotor (Beckman) for 45 min at 4°C and purified by affinity chromatography with amylose resin (New England Biolabs). Amylose resin with bound MBP-GLIC was washed with 10 volumes of B1 with 0.1% DDM followed by 10 volumes of B1 with 0.02% DDM (buffer B2), and then the fusion protein was eluted in B2 supplemented with 20 mM maltose. MBP-GLIC was concentrated in Amicon Ultra-4 (100 KDa molecular weight cutoff) concentrator tubes (Millipore) and subjected to size exclusion gel filtration in a Superose6 GL10/300 column (GE Healthcare) previously equilibrated in B2. Fractions of the peak corresponding to pentameric MBP-GLIC (∼400 kDa) were combined and treated with MTSL and thrombin under gentle agitation at 4°C overnight. In detail, protein was first treated with 5-fold molar excess of DTT for 5 min at room temperature, and then 2- to 60-fold molar excess of MTSL (Toronto Research) was added to specifically label the unique cysteines, followed by 1 U of thrombin (bovine, plasminogen-free, Calbiochem) per 100 µg of pentameric MBP-GLIC. The digested product was applied to amylose resin for a second round of affinity chromatography to purify the cleaved, MTSL-labeled GLIC from the excess spin label and MBP. GLIC was subjected to a final gel filtration, and peak fractions corresponding to the pentameric form of the protein (∼180 kDa) were combined and concentrated to 3–6 mg/mL, flash frozen in liquid nitrogen, and stored at −80°C.

### Reconstitution of Purified GLIC Into Liposomes

Purified GLIC protein was reconstituted into liposomes formed with 1-palmitoyl-2-oleoyl-sn-glycero-3-phosphoethanolamine (PE) and 1-palmitoyl-2-oleoyl-sn-glycero-3-phospho-(1′-rac-glycerol) (PG) at a PE∶PG = 2.7∶1 molar ratio, or with PE∶PG∶cholesterol at a 3.4∶1.3∶1 molar ratio. Lipid mixtures were prepared at a concentration of 20 mg/mL in buffer B1, sonicated, and mixed with GLIC purified in DDM, typically at 6,000-fold molar excess. After a 3 h incubation at 4°C, the protein∶lipid mixture was diluted 2-fold in buffer B1 containing 10% glycerol and incubated overnight at 4°C. To remove DDM, Biobeads (BioRad) were added for 8–10 h and then removed. Finally, the Biobead-free solution was ultracentrifuged at 100,000 rpm, and the pellets of GLIC reconstituted into liposomes were stored at −80°C.

### Two-Electrode Voltage Clamp Recording of *Xenopus leavis* Oocytes Injected with Purified GLIC Reconstituted Into Liposomes

Pellets of purified GLIC mutants reconstituted into liposomes were thawed on ice. The amount of protein in a pellet was estimated by assuming 70% reconstitution efficiency. The pellets were resuspended in buffer B1 to a protein concentration of approximately 1–2 mg/mL and were subjected to two rounds of freeze-thaw. The proteoliposome solution was somewhat viscous and to facilitate protein injection into the oocytes, the diameter of the glass injection pipet was adjusted to about 5–10 µm. Protein-injected oocytes were incubated for 5–8 h at 16°C before recording. Two-electrode voltage clamp of oocytes injected with lipid reconstuted GLIC protein was performed in the same manner as oocytes injected with GLIC cRNA. pH-induced currents from uninjected oocytes were used as controls.

### Single-Channel Recordings in Planar Lipid Bilayers

For preparation of planar lipid bilayers, lipid mixtures of PE∶PG (2.7∶1), PE∶PG∶cholesterol (3.4∶1.3∶1), or PE∶PG∶cardiolipin (5.8∶2.3∶1) were prepared in n-decane at a concentration of 20 mg/mL. Planar lipid bilayers were painted with a glass rod across a 150 µm aperture in a Delrin cup. In order to create both an ionic and a pH gradient, the *trans* chamber was filled with 150 mM NaCl at pH 7.6, whereas the *cis* chamber (where the protein was added to) was filled with 450 mM NaCl at pH 5.2. Prior to adding the GLIC K32R1, T157R1, and P249R1 protein to the chamber, the protein was treated with 10 mM dithiothreitol (DTT) to remove the majority of the spin label. Once the protein was incorporated into the planar lipid bilayer, the pH of the *cis* chamber was dropped to 4.6 by adding 10% (v∶v) of 1 M Na Citrate. Single-channel currents were recorded using an Axopatch 200B amplifier (Axon Instruments), filtered with an 8-pole low-pass Bessel filter (Frequency Devices) set at 100 Hz, and digitized at a rate of 4 kHz with a Digidata 1440A interface (Axon Instruments). Data acquisition and analysis were performed with pClamp10.2.

### EPR Spectroscopy

Continuous wave (CW) EPR spectroscopy was carried out at room temperature on a Bruker ELEXSYS 500 X-band spectrometer equipped with a superhigh Q (SHQ) cavity (Bruker Biospin). Upon change in pH to 4.6 or 3.0, the proteolipid samples were freeze-thawed to ensure even distribution of the protons inside and outside the vesicles. Spectra were then recorded over 100 G under nonsaturating conditions with a 100 kHz field modulation of 1 G. Samples were typically 20 µL in volume and contained in a glass capillary. Protein concentrations were typically 30 µM.

The DEER spectroscopy experiments were carried out at the Ohio Advanced EPR Laboratory at Miami University using a Bruker ELEXSYS 580 Q-band spectrometer equipped with a Bruker EN5107D2 dielectric resonator or at the National Biomedical EPR Center using a Bruker ELEXSYS 580 X-band spectrometer equipped with a Bruker 3 mm split-ring cavity. Samples were typically 10 µL for Q-band and 25 µL for X-band at a concentration of 35–50 µM, contained 20% deuterated glycerol as a cryoprotectant, were flash frozen using a dry ice-acetone slurry, and run at 80 K. A four-pulse DEER sequence [42] was used with two-step phase cycling. The dipolar evolution data were analyzed for distance distributions using DeerAnalysis2011 software [62] and model-free Tikhonov regularization as it gave the best fit to the background-corrected data. Distribution curves obtained from model-free Tikhonov regularization were then fit to Gaussian shapes using Peak Fitter (T. O'Haver, MATLAB File Exchange) to obtain the mean peak center distance values. Rice3D and Gaussian analyses of the dipolar evolution data yielded similar results as the model-free Tikhonov regularization analysis. All DEER data distributions shown are the result of model-free Tikhonov regularization. Pairs of data were recorded on the same spectrometers and under identical conditions.

### Calculation of Minimum Displacement δ

The minimum displacement δ for a spin probe is calculated using the formula:
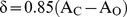
(1a)or
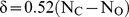
(1b)where A_C_ and A_O_ are the DEER-determined distances between probes in adjacent subunits (indicated as “short” in Table 1) at pH 7.6 and 4.6, respectively, and N_C_ and N_O_ are the distances between probes in nonadjacent subunits (indicated as “long” in Table 1) at pH 7.6 and 4.6, respectively (see Figure S4B). The formulas are also used to calculate the displacement of a residue in GLIC relative to the structurally aligned amino acid in ELIC using the crystal structures. In this case, A_C_ and A_O_ are the C_β_–C_β_ distances separating pairs of equivalent residues in adjacent subunits in ELIC and GLIC, respectively, whereas N_C_ and N_O_ are the distances separating pairs of equivalent residues in nonadjacent subunits in ELIC and GLIC, respectively. In this method the displacement δ depends on distances calculated within each crystal structure (i.e., the intrinsic coordinates), which is a more reliable method than positioning the two structures on top of each other and measuring distances between aligned residues.

### Homology Modeling and Computational Modeling of MTSL on GLIC

A closed state homology model of GLIC based on the crystal structure of ELIC (PDB entry 2VL0) was built using Modeller as described by Ghosh and co-workers [63]. The PRONOX program (http://rockscluster.hsc.usc.edu/research/software/pronox/pronox.html) was used as described by Hatmal and colleagues [64] to estimate the distances between spin labels using our GLIC homology model and the crystal structure of GLIC (PDB entry 3EAM) as inputs. In general, the PRONOX distances were estimated using the standard approach. For some positions, we used the fine search option to help remove clashes. All distances are from N to N atoms. The computed PRONOX distances calculated for the GLIC homology model and the GLIC crystal structure were compared to our experimental DEER distances measured at pH 7.6 and pH 4.6, respectively. Note, the DEER distances are obtained from lipid-embedded functional GLIC protein, while the PRONOX distances are based on static X-ray crystal structures of two different proteins in detergent micelles.

## Supporting Information

Figure S1 **Functional characterization of GLIC mutants in oocytes.** (A) Chemical structure of MTSL and the R1 side chain that is created upon reaction of MTSL with cysteine. (B) pH dose-response curves for wild-type and mutant GLIC receptors expressed in *Xenopus laevis* oocytes. All mutants formed functional channels. (C) Representative currents induced by pH 5.0 buffer from oocytes expressing C26A and T157C before and after 2 min application of 100 µM and 1 µM MTSL, respectively. MTSL significantly reduced proton-mediated current amplitude for T157C, indicating that MTSL covalently modified the introduced cysteine at this position.
(TIFF)

Figure S2 **CW EPR spectrum from MTSL-treated C26A GLIC mutant receptor.** No significant EPR signal is observed, indicating the absence of spin-labeled protein contaminants. (Left) Expanded view of GLIC crystal structure with C26 shown in space-fill.
(TIFF)

Figure S3 **CW EPR spectra of K247R1 and P249R1 GLIC mutant receptors.** Comparisons of CW EPR spectra of K247R1 (top right) and P249R1 (bottom right) GLIC mutants reconstituted into PE∶PG liposomes at pH 7.6 (black), pH 4.6 (blue), and pH 3.0 (red). For K247R1, pH 3.0 induced an additional slight decrease in probe mobility compared to pH 4.6. Expanded view of GLIC crystal structures are shown with spin-labeled positions K247 (top left) and P249 (bottom left) in space-fill.
(TIFF)

Figure S4 **Calculating spin probe displacement, δ.** (A) Because GLIC is a homopentamer, two distances are expected at each pH: one between spin probes on adjacent subunits, another between probes on nonadjacent subunits. (B) Schematic diagram illustrating the proton-induced displacement, δ, of the spin probe in a single subunit based on the DEER data. A_C_ and A_O_ are the DEER-determined distances for adjacent subunits at pH 7.6 and 4.6, respectively; N_C_ and N_O_ are the distances for nonadjacent subunits at pH 7.6 and 4.6, respectively; r_C_ and r_O_ are the radii of circles circumscribing the pentagons. To take into account a general quaternary twisting, δ is derived assuming a rotation θ of one pentagon relative to the other. For simplicity, we assume the two pentagons lie on the same plane. The resulting equation can be derived using basic geometry and trigonometry. The simplest case, no rotation (i.e., θ = 0), provides the minimum displacement a spin probe undergoes with activation.
(TIFF)

Table S1 **Summary of pH responses and MTSL modification of WT and mutant GLIC channels.** pH_50_ is pH value that elicited 50% of the maximal proton-induced current. n_H_ is the Hill coefficient. Data are mean ± SEM from *n* experiments. MTSL modification is defined as (1−I_after MTSL_/I_before MTSL_)*100%, where I_after MTSL_ and I_before MTSL_ are currents elicited by pH_50_ proton concentration after and before exposure to MTSL, respectively. Data are mean ± SEM from n2 experiments. Values significantly different from C26A, *p<0.01, **p<0.001.
(DOC)

Table S2 **Intersubunit distances estimated by PRONOX (** http://rockscluster.hsc.usc.edu/research/software/pronox/pronox.html **) between spin labels placed in a GLIC homology model based on the ELIC crystal structure (PDB entry 2VL0) and in the GLIC crystal structure (PDB entry 3EAM).** ND: Using standard conditions in the program, no distances were computed for C26R1, T157R1, and P249R1 due to clashes (i.e., PRONOX could not place MTSL at the position). *Using relaxed conditions, Pronox could still not place MTSL at these positions and no distances were computed. **Using relaxed conditions, intersubunit distances of 25 Å (adjacent) and 40 Å (nonadjacent) for T157R1 were estimated.
(DOC)

## Abbreviations

ELIC: *Erwinia chrysanthemi* ligand-gated ion channel
GLIC: *Gloeobacter violaceus* ligand-gated ion channel
MTSL: 1-oxyl-2,2,5,5-tetramethyl-3-pyrroline-3-methyl methanethiosulfonate spin label
pLGICs: pentameric ligand-gated ion channels
SDSL EPR: site-directed spin labeling electron paramagnetic resonance
