# Supplementary figures and images for: Site-Directed Spin Labeling Reveals Pentameric Ligand-Gated Ion Channel Gating Motions
Source: PLoS Biol. 2013 Nov 19;11(11):e1001714. doi: 10.1371/journal.pbio.1001714 (PMC3833874; doi:10.1371/journal.pbio.1001714)

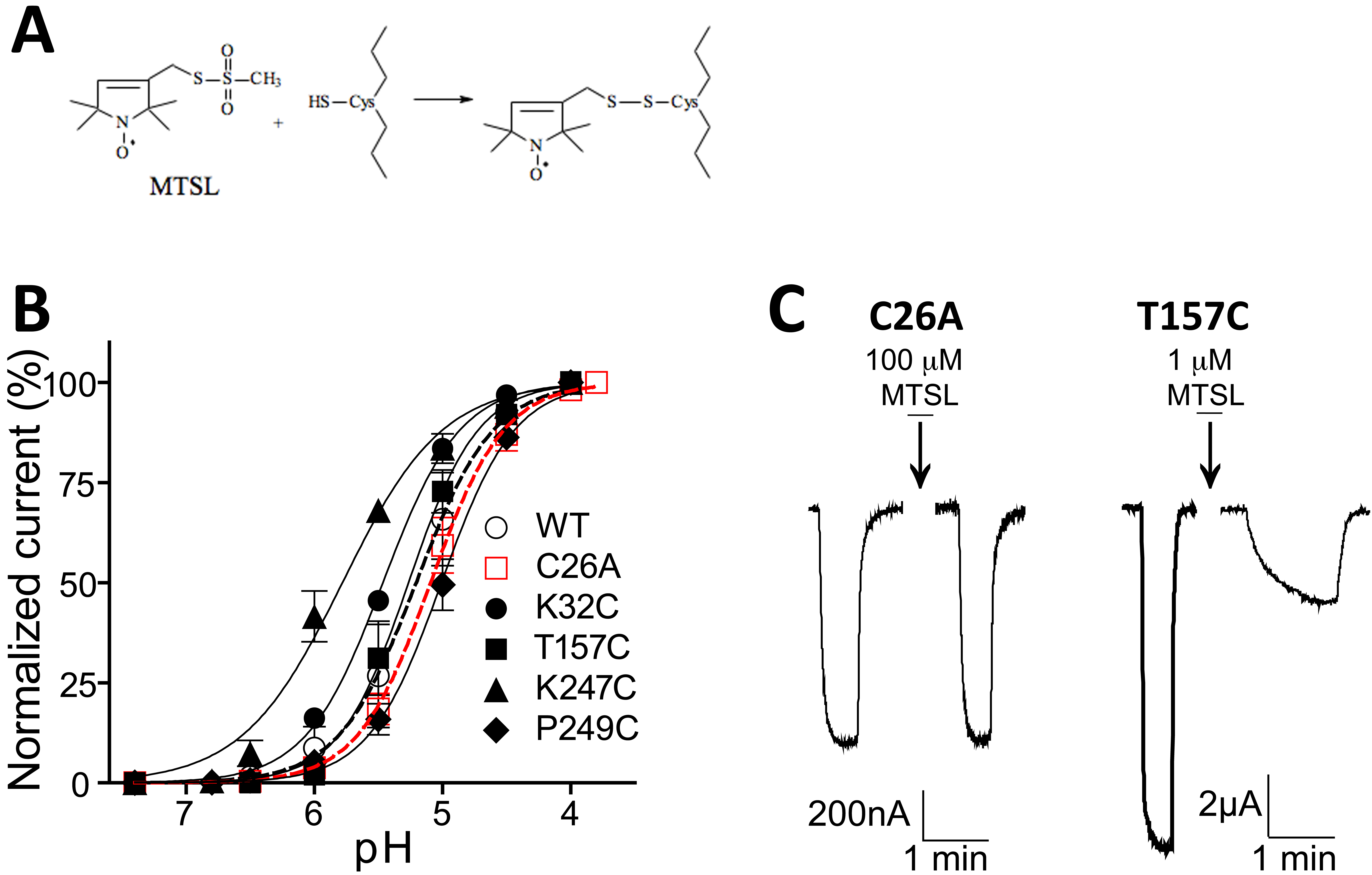

Supplement: Figure S1 — Functional characterization of GLIC mutants in oocytes. (A) Chemical structure of MTSL and the R1 side chain that is created upon reaction of MTSL with cysteine. (B) pH dose-response curves for wild-type and mutant GLIC receptors expressed in Xenopus laevis oocytes. All mutants formed functional channels. (C) Representative currents induced by pH 5.0 buffer from oocytes expressing C26A and T157C before and after 2 min application of 100 µM and 1 µM MTSL, respectively. MTSL significantly reduced proton-mediated current amplitude for T157C, indicating that MTSL covalently modified the introduced cysteine at this position. (TIFF) [file pbio.1001714.s001.tif]

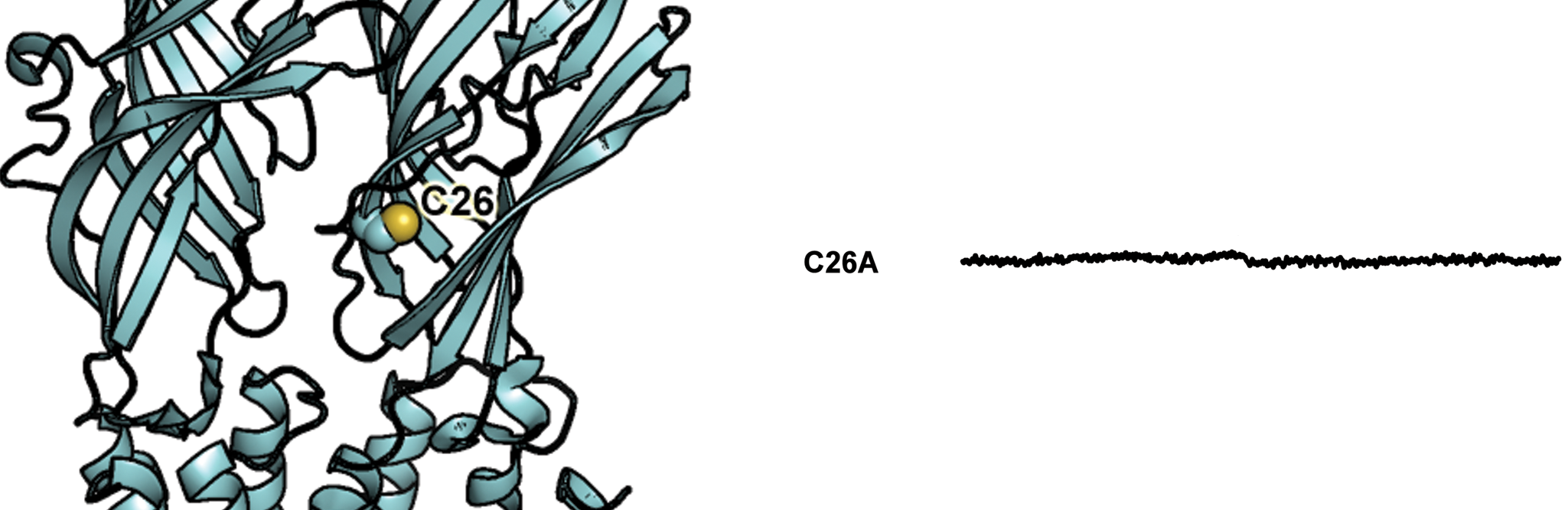

Supplement: Figure S2 — CW EPR spectrum from MTSL-treated C26A GLIC mutant receptor. No significant EPR signal is observed, indicating the absence of spin-labeled protein contaminants. (Left) Expanded view of GLIC crystal structure with C26 shown in space-fill. (TIFF) [file pbio.1001714.s002.tif]

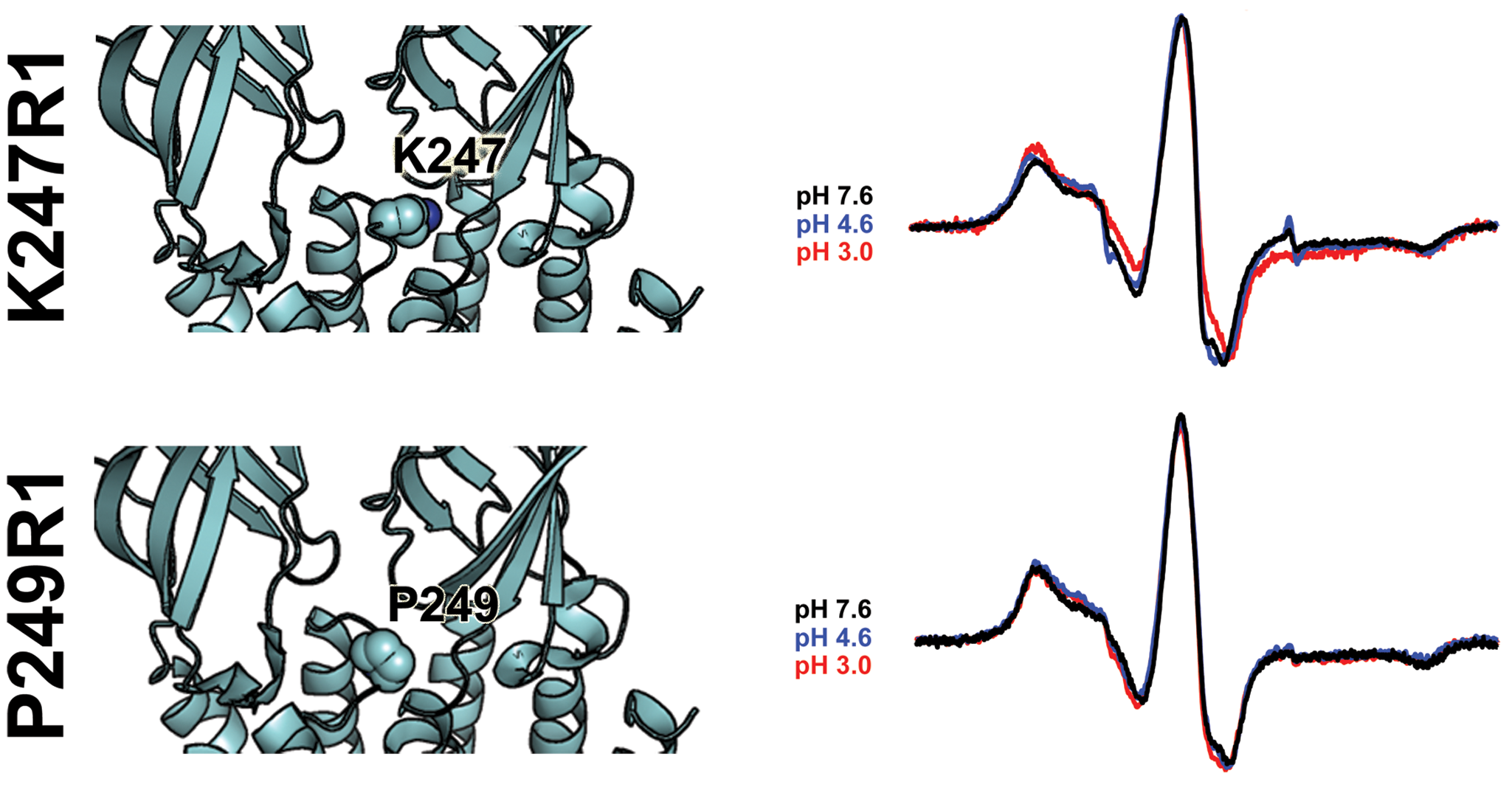

Supplement: Figure S3 — CW EPR spectra of K247R1 and P249R1 GLIC mutant receptors. Comparisons of CW EPR spectra of K247R1 (top right) and P249R1 (bottom right) GLIC mutants reconstituted into PE∶PG liposomes at pH 7.6 (black), pH 4.6 (blue), and pH 3.0 (red). For K247R1, pH 3.0 induced an additional slight decrease in probe mobility compared to pH 4.6. Expanded view of GLIC crystal structures are shown with spin-labeled positions K247 (top left) and P249 (bottom left) in space-fill. (TIFF) [file pbio.1001714.s003.tif]

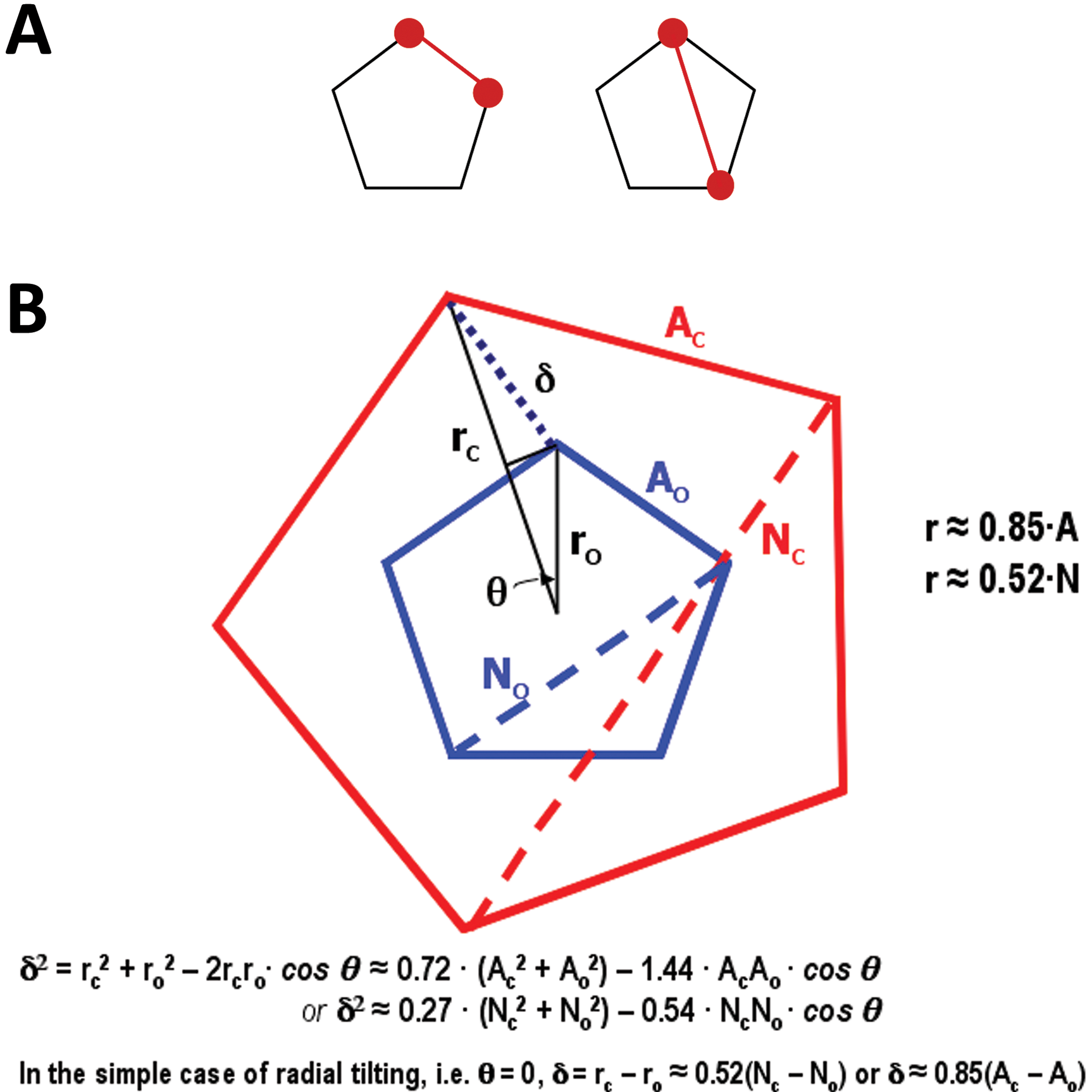

Supplement: Figure S4 — Calculating spin probe displacement, δ. (A) Because GLIC is a homopentamer, two distances are expected at each pH: one between spin probes on adjacent subunits, another between probes on nonadjacent subunits. (B) Schematic diagram illustrating the proton-induced displacement, δ, of the spin probe in a single subunit based on the DEER data. AC and AO are the DEER-determined distances for adjacent subunits at pH 7.6 and 4.6, respectively; NC and NO are the distances for nonadjacent subunits at pH 7.6 and 4.6, respectively; rC and rO are the radii of circles circumscribing the pentagons. To take into account a general quaternary twisting, δ is derived assuming a rotation θ of one pentagon relative to the other. For simplicity, we assume the two pentagons lie on the same plane. The resulting equation can be derived using basic geometry and trigonometry. The simplest case, no rotation (i.e., θ = 0), provides the minimum displacement a spin probe undergoes with activation. (TIFF) [file pbio.1001714.s004.tif]
