# Supplementary material for: Site-Directed Spin Labeling Reveals Pentameric Ligand-Gated Ion Channel Gating Motions
Source: PLoS Biol. 2013 Nov 19;11(11):e1001714. doi: 10.1371/journal.pbio.1001714 (PMC3833874; doi:10.1371/journal.pbio.1001714)
Supplement: Table S1 — Summary of pH responses and MTSL modification of WT and mutant GLIC channels. pH50 is pH value that elicited 50% of the maximal proton-induced current. nH is the Hill coefficient. Data are mean ± SEM from n experiments. MTSL modification is defined as (1−Iafter MTSL/Ibefore MTSL)*100%, where Iafter MTSL and Ibefore MTSL are currents elicited by pH50 proton concentration after and before exposure to MTSL, respectively. Data are mean ± SEM from n2 experiments. Values significantly different from C26A, *p<0.01, **p<0.001. (DOC) [file pbio.1001714.s005.doc]

**Supplementary Table 1.**

|  | pH_50_ | n_H_ | n | MTSL modification (%) | n2 |
| --- | --- | --- | --- | --- | --- |
| WT | 5.2±0.1 | 1.6±0.1 | 6 | 5.1 ± 3.1 | 4 |
| C26A | 5.1±0.1 | 1.7±0.1 | 8 | 9.1 ± 5.4 | 5 |
| K32C | 5.5±0.2 | 1.5±0.1 | 3 | -29.7 ± 2.3** | 3 |
| T157C | 5.3±0.1 | 1.9±0.2 | 10 | -63.0 ± 2.1** | 3 |
| K247C | 5.8 ± 0.1** | 1.3 ± 0.1* | 8 | 3.0 ± 8.0 | 2 |
| P249C | 5.0±0.1 | 1.6±0.1 | 9 | -73.5 ± 1.5** | 2 |
