# Supplementary material for: Site-Directed Spin Labeling Reveals Pentameric Ligand-Gated Ion Channel Gating Motions
Source: PLoS Biol. 2013 Nov 19;11(11):e1001714. doi: 10.1371/journal.pbio.1001714 (PMC3833874; doi:10.1371/journal.pbio.1001714)
Supplement: Table S2 — Intersubunit distances estimated by PRONOX ( http://rockscluster.hsc.usc.edu/research/software/pronox/pronox.html ) between spin labels placed in a GLIC homology model based on the ELIC crystal structure (PDB entry 2VL0) and in the GLIC crystal structure (PDB entry 3EAM). ND: Using standard conditions in the program, no distances were computed for C26R1, T157R1, and P249R1 due to clashes (i.e., PRONOX could not place MTSL at the position). *Using relaxed conditions, Pronox could still not place MTSL at these positions and no distances were computed. **Using relaxed conditions, intersubunit distances of 25 Å (adjacent) and 40 Å (nonadjacent) for T157R1 were estimated. (DOC) [file pbio.1001714.s006.doc]

|  | **GLIC “closed” modeled on ELIC** | | **GLIC (pdb: 3EAM)** | |
| --- | --- | --- | --- | --- |
|  | adjacent (Å) | non adjacent (Å) | adjacent (Å) | non adjacent (Å) |
| **C26R1** | ND * | ND | ND * | ND |
| **K32R1** | 14 | 22.4 | 10 | 16 |
| **T157R1** | ND ** | ND | 17 | 27.2 |
| **K247R1** | 7.8 | 12.4 | 16 | 25.6 |
| **P249R1** | ND * | ND | 36 | 57.6 |

**Supplementary Table 2.**
